# Supplementary material for: Direct Photochemical Synthesis of Substituted Benzo[b]fluorenes
Source: Org Lett. 2024 Nov 25;26(48):10364–8. doi: 10.1021/acs.orglett.4c03978 (PMC11629382; doi:10.1021/acs.orglett.4c03978)
Supplement: Supplementary file 2 — ol4c03978_si_002.zip [file ol4c03978_si_002.zip › FID for Publication/20240705_RC-2-C-4-81B_01/plots/RC-2-C-4-81B_gHSQCAD_20240707_01_plot01.pdf]

Sample Name:
RC-2-C-4-81B
Data Collected on:
newucd400-vnmrs400
Archive directory:
/home/data/MBaumann/ucd400/RuairiCrawford/2024
Sample directory:
20240705_RC-2-C-4-81B_01
FidFile: RC-2-C-4-81B_gHSQCAD_20240707_01

Pulse Sequence: gHSQCAD
Solvent: cdcl3
Data collected on: Jul 7 2024

Temp. 25.0 C / 298.1 K
Sample #22, Operator: MBaumann

Relax. delay 1.000 sec
Acq. time 0.150 sec
Width 3980.9 Hz
2D Width 17094.0 Hz
4 repetitions
2 x 256 increments
OBSERVE H1, 399.8783237 MHz
DECOUPLE C13, 100.5570260 MHz
Power 34 dB
on during acquisition
off during delay
W40_P8012 modulated
DATA PROCESSING
Gauss apodization 0.069 sec
F1 DATA PROCESSING
Gauss apodization 0.014 sec
FT size 2048 x 2048
Total time 41 min

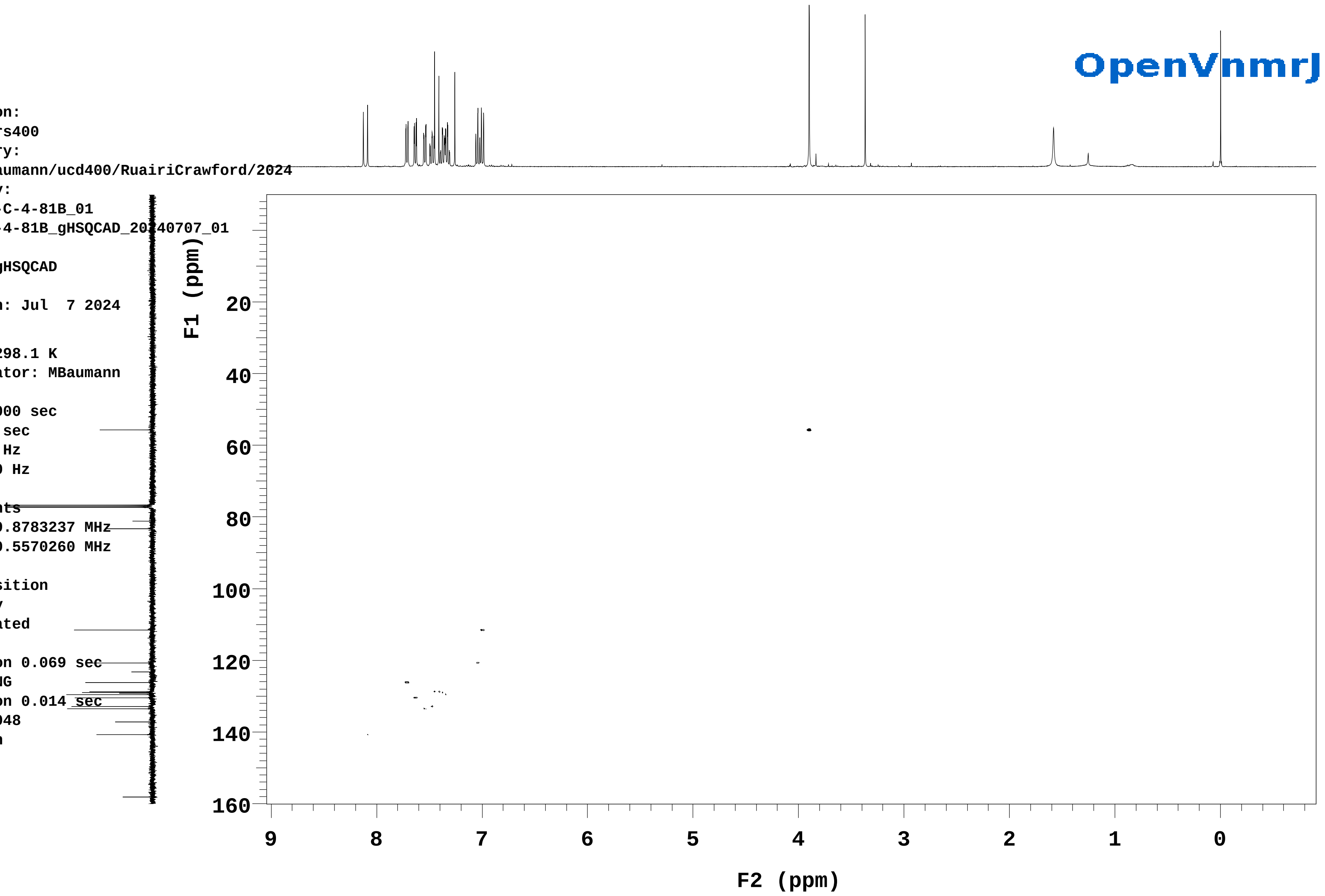


Plotname: RC-2-C-4-81B_gHSQCAD_20240707_01_plot01
